# An Investigation of Novel Series of 2-Thioxo-1,3-dithiol-carboxamides as Potential Antispasmodic Agents: Design, Synthesis via Coupling Reactions, Density Functional Theory Calculations, and Molecular Docking

**DOI:** 10.3390/molecules29163855

**Published:** 2024-08-14

**Authors:** Riham Sghyar, Mouad Lahyaoui, Noura Aflak, Oussama Moussaoui, Alae Chda, Rachid Bencheikh, El Mestafa El Hadrami, Nada Kheira Sebbar, Ashwag S. Alanazi, Mohamed Hefnawy

**Affiliations:** 1 Laboratory of Applied Organic Chemistry, Faculty of Science and Techniques, Sidi Mohamed Ben Abdellah University, Routed ‘Imouzzer, B.P. 2202, Fez 30050, Moroccoelmestafa.elhadrami@usmba.ac.ma (E.M.E.H.); 2 Department of Chemistry, Faculty of Science, University Ibn Zohr, Agadir 80000, Morocco; 3 Laboratory of Analytical and Molecular Chemistry/LAMC, Polydisciplinary Faculty, Cadi Ayyad University, Safi 46030, Morocco; 4 Laboratory of Organic Synthesis, Extraction and Valorization, Department of Chemistry, Faculty of Sciences Ain Chock, Hassan II University of Casablanca, B.P. 5366, Casablanca 20670, Morocco; 5 Institut Supérieur Des Professions Infirmières Et Techniques de Santé, Rabat 60000, Morocco; 6 Microbial Biotechnology and Bioactive Molecules Laboratory, FST, USMBA, B.P. 2202, Fez 30050, Morocco; 7 Applied Chemistry and Environment Laboratory, Applied Bioorganic Chemistry Team, Faculty of Science, Ibn Zohr University, Agadir 80000, Morocco; 8 Laboratory of Heterocyclic Organic Chemistry, Drug Science Research Center, Pharmacochemistry Competence Center, Faculty of Sciences, Mohammed V University in Rabat, Av. Ibn Battouta, B.P. 1014, Rabat 10000, Morocco; 9 Department of Pharmaceutical Sciences, College of Pharmacy, Princess Nourah bint Abdulrahman University, Riyadh 11671, Saudi Arabia; 10 Department of Pharmaceutical Chemistry, College of Pharmacy, King Saud University, Riyadh 11451, Saudi Arabia

**Keywords:** peptidic coupling, amino acids, 2-thioxo-1,3-dithiol-carboxamides (**TDTCAs**), antispasmodic activity, molecular docking, DFT Calculations

## Abstract

This study reports the synthesis of 2-thioxo-1,3-dithiol-carboxamides (**TDTCAs**) under mild conditions at room temperature using HBTU as a coupling agent, which significantly improved amide bond formation. The synthesized compounds were characterized using several analytical techniques, including ^1^H and ^13^C NMR spectroscopy, and HRMS, confirming their intended structures and structural integrity. A DFT computational study at the B3LYP/6-31G(d,p) level was conducted on the four synthesized compounds to compare their electronic properties and molecular structures. The results showed that these compounds demonstrated antispasmodic effects on jejunum contractions. Molecular docking revealed that compounds **c** and **d** displayed the highest docking scores on potassium and voltage-gated calcium channels and adrenergic receptors. In summary, compounds **c** and **d** exhibit antispasmodic effects, potentially blocking alpha-adrenergic receptors and calcium channels, thus providing a scientific basis for their potential use in treating gastrointestinal disorders.

## 1. Introduction

**Scheme 1:**
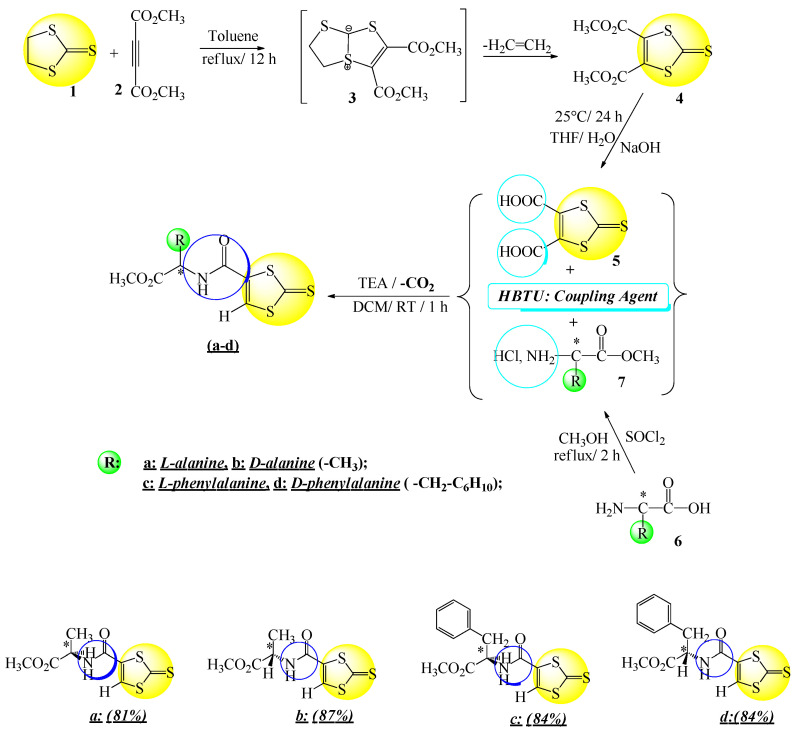
General synthetic route of the target of 2-thioxo-1,3-dithiol-carboxamides (**a**–**d**). The yields presented are the isolated yields after purification in all cases.

The sulfur-heterocycles are found in a variety of natural products, and biological systems cannot ignore these compounds as effective pharmacophores. An intriguing factor is that these compounds are markedly more spasmolytic than their counterparts [1,2,3,4,5,6,7,8,9]. Researchers have been particularly interested in 1,3-dithiole-2-thione due to its exceptional sulfur redox chemistry and polarizability [10,11,12]. These compounds have been applied to a number of materials science applications [13,14], notably for creating new conductive materials. A major challenge in designing bioactive molecules is their pharmacokinetic properties, which include solubility, bioavailability, and specificity of their biological interactions. By coupling 1,3-dithiole-2-thiones with different amino acids, these properties can be improved because amino acids possess many biological properties and are highly soluble in water [15,16,17,18], possibly enhancing the biological activities of the two components, which may arouse the interest of new bioactive molecules. As shown in Scheme 1, the aim is to couple 1,3-dithiole-2-thiones with amino acids, namely, L-/D-alanine and L-/D-phenylalanine, which can be achieved via amide bond-forming reactions. Amino acids can be protected to avoid non-specific reactions, and coupling agents such as HBTU can be used to activate the carboxyl groups of 1,3-dithiole-2-thiones for amide bond formation. 

As shown in Scheme 1, the aim is to couple 1,3-dithiole-2-thiones with amino acids namely L-/D-alanine and L-/D-phenylalanine, which can be achieved via amide bond-forming reactions. Amino acids can be protected to avoid non-specific reactions, and coupling agents such as HBTU can be used to activate the carboxyl groups of 1,3-dithiole-2-thiones for amide bond formation. The structural and electronic analysis of the synthesized products were studied by the density functional theory (DFT) approach at the B3LYP/6−31G(d,p) level, the results of which indicated that the compound **d** was more favorable. The synthetic compounds were evaluated for their potential as spasmolytic candidates in vitro using isolated tissues from animals. In this study, we report the antispasmodic effects of a novel series of 2-thioxo-1,3-dithiol-carboxamides on the intestinal contractile activity of rabbit jejunum. Our findings indicate that some molecules of 2-thioxo-1,3-dithiol-carboxamides, specifically compounds **c** and **d**, have the potential to bind to adrenergic receptors and voltage-gated calcium channels, as suggested by molecular docking analyses in silico.

## 2. Results and Discussion 

### 2.1. Synthesis of 2-Thioxo-1,3-dithiol-carboxamides (TDTCAs)

The primary objective of this research is to synthesize a novel series of 2-thioxo-1,3-dithiol-carboxamides (**TDTCAs**) (referred to as compounds **a**–**d**). The initial stage of our work involved a two-step process. Initially, we examined the synthesis of 2-thioxo-1,3-dithiol-4,5-dicarboxylic acid (**5**), a crucial intermediate in organic synthesis, particularly in coupling reactions. To synthesize dimethyl 2-thioxo-1,3-dithiol-4,5-dicarboxylate, the reaction between ethylene trithiocarbonate and dimethyl acetylenedicarboxylate (DMAD, compound **2**) needs to be carried out by refluxing in toluene, leading to a substantial yield of dimethyl 2-thioxo-1,3-dithiol-4,5-dicarboxylate (compound **4**) [19]. The process is strategically advantageous, and it is proposed to be proceeded by a crucial 1,3-dipolar cycloaddition, leading to the formation of an intermediate ylide (compound **3**). The formation of compound **4** is then facilitated by a retro-1,3-dipolar cycloaddition, as illustrated in Scheme 1 and elucidated in the proposed mechanism (formula 1). Furthermore, under alkaline conditions and in the presence of water, (compound **4**) undergoes hydrolytic cleavage to give (compound **5**) [19]. The progression of the reaction was monitored through thin-layer chromatography (TLC). The resulting synthesized compounds were purified via column chromatography using silica gel as the stationary phase, resulting in a 91% yield for the acidic compound as illustrated in Scheme 1. The next phase of the process involved the conversion of four amino acids into their respective methylated amino esters. This conversion was achieved by treating the amino acids with thionyl chloride (SOCl_2_) in refluxing methanol [20,21,22,23]; the desired result is the formation of methyl ester groups (L-alanine-OMe, D-alanine-OMe, L-phenylalanine-OMe, L-phenylalanine-OMe).

The final step was the reaction between the substrate 2-thioxo-1,3-dithiol-4,5-dicarboxylic acid (**5**) and the four methylated amino ester hydrochloric acid salts (L-alanine-OMe, D-alanine-OMe, L-phenylalanine-OMe, D-phenylalanine-OMe). It takes an hour for the reaction to complete at room temperature, facilitated by hexafluorophosphate benzotriazole tetramethyl uronium (HBTU) as a coupling agent, in a basic medium with triethylamine (TEA) and dichloromethane (DCM) as suitable solvents, while maintaining ideal conditions [20,21,22,23]. Compounds (**a**–**d**) synthesized in (Scheme 1) were purified by liquid chromatography with silica gel as the stationary phase, yielding 81% to 89%. The confirmation of their chemical structures was established through analysis by both ^1^H and ^13^C NMR spectroscopy, as well as high-resolution mass spectra (HRMS), adhering to scientific methodologies in the field of organic synthesis and chemical analysis.

Compounds (**a**–**d**) were characterized by ^1^H, ^13^C NMR spectroscopy and ESI-TOF mass-spectrometry. As an example, the ^1^H NMR spectrum of compound **a** revealed the presence of a doublet assigned to the methyl group of alanine CH_3_ proton CH at δ = 1.5 ppm with a coupling constant *J* = 7.2 Hz, while two singlet signals at δ = 3.8 ppm and δ = 6.7 ppm illustrated the presence of the protons of CO_2_-CH_3_ and HCS, respectively. In addition, the protons quadruplet CH appear in the form of a multiplet at δ = 4.6. The ^13^C NMR spectra indicate the signals of methyl groups of alanine, peptide amide, the ester group and CH group of 2-thioxo-1,3-dithiol-carboxamides derivatives at δ = 18.64 ppm, δ = 49.20 ppm, δ = 49.20, and 132.52 ppm, respectively, while two quaternary carbons, CO and CSO, appear at 53.36 ppm and 157.33 ppm, respectively. A signal of quaternary carbon C-CO_2_ resonated at δ = 173.53 ppm, whereas the signal of quaternary carbon Cq=S appears at δ = 211.11 ppm, confirming the presence of the 2-thioxo-1,3-dithiol- derivatives containing carboxamides compounds (**TDTCAs**). The ESI-TOF mass spectrum of compound **a** showed [MH]+ peak at *m*/*z* = 263, which is in agreement with its molecular formula C_8_H_9_NO_3_S. The synthesis of these compounds was confirmed using a comprehensive range of spectroscopic techniques.

### 2.2. Plausible Mechanism of the Coupling Reaction by HBTU 

**Scheme 2:**
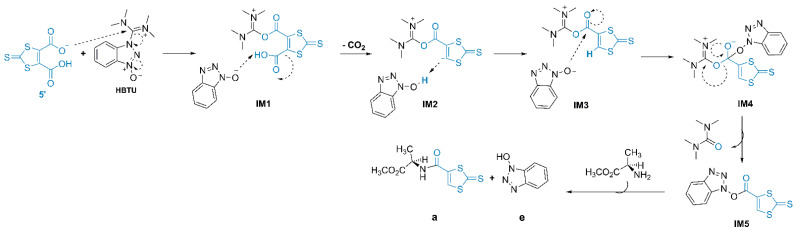
Suggested mechanism of the coupling reaction between 2-Thioxo-1,3-dithiol-4,5-dicarboxylic acid 5 and protected L-alanine by HBTU agent.

In order to elucidate a precise understanding of the reaction mechanism of the coupling reaction by the HBTU agent between 2-Thioxo-1,3-dithiol-4,5-dicarboxylic acid **(5)** and protected L-alanine, a detailed description of the proposed mechanism based on literature data is depicted in Scheme 2 [24]. Initially, HBTU interacts with the deprotonated 2-Thioxo-1,3-dithiol-4,5-dicarboxylic acid **5′** facilitated by triethylamine (TEA). This interaction results in the formation of intermediate IM1, which subsequently decarboxylates in the presence of HBTU to give intermediate IM3. The formation of the final product then follows the usual mechanism for the formation of the amide bond with this coupling reagent (Scheme 2). 

### 2.3. Structural and Electronic Properties of the Synthesized Compounds 

**Figure 1:**
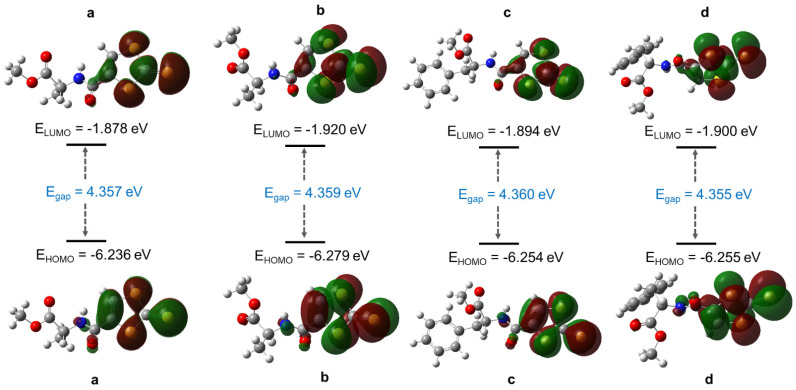
The optimized structures and the HOMO-LUMO of compounds (**a**–**d**) at the B3LYP/6-31G(d,p) level.

To comprehend the reactivity at the atomic level and the chemical stability of molecules, the geometric structure of synthesized 2-thioxo-1,3-dithiol-carboxamides **(TDTCAs)** (**a**–**d**) was optimized via DFT approach using the B3LYP/6-31G(d,p) method (Figure 1). In this regard, the HOMO-LUMO energy gap was calculated, and the results are summarized in Figure 1. It shows that the HOMO and LUMO are localized in the region of the 2-thioxo-1,3-dithiol, which makes this region of the molecule more reactive. 

**Figure 2:**
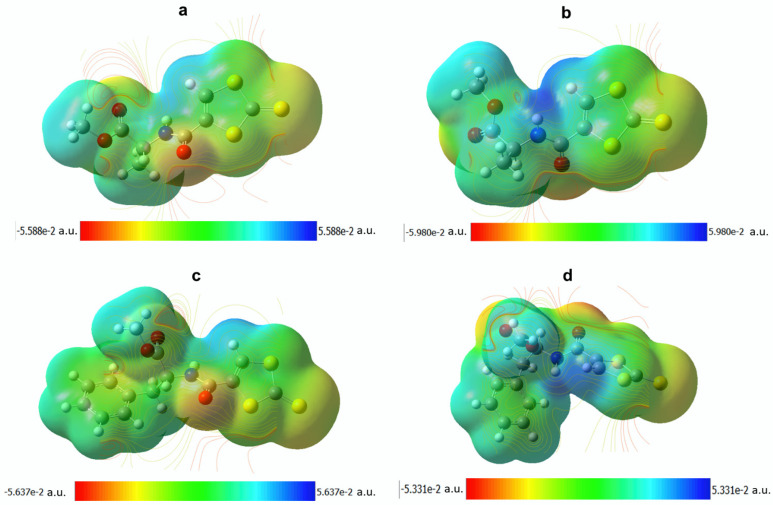
The Molecular electrostatic potential (MEP) in atomic units (a.u.) and their contours of the compounds (**a**–**d**).

In order to visualize electrophilic/nucleophilic sites in synthesized compounds, the molecular electrostatic potential (MEP) calculations were investigated as a powerful tool in this approach (Figure 2) [25]. In the MEP maps, high electronic density is represented by the color red, while low electronic density is represented by the color blue. Red regions correspond to nucleophile sites, whereas blue regions correspond to electrophile sites. Therefore, the red and blue regions are assigned as the most suitable sites for nucleophilic and electrophilic attacks, respectively [25]. The MEP maps revealed that more nucleophilic sites exist on the oxygen and sulfur atoms, while the electrophilic sites are present on the hydrogen atom attached to the nitrogen atom of the amide, as well as on the hydrogen atom in the region of the 2-thioxo-1,3-dithiol of all the synthesized compounds. 

### 2.4. Antispasmodic Activity

#### Effect of 2-Thioxo-1,3-dithiol Derivatives Containing Carboxamides Compounds (TDTCAs) on ACh Induced Contractions of Rabbit Jejunum

**Figure 3:**
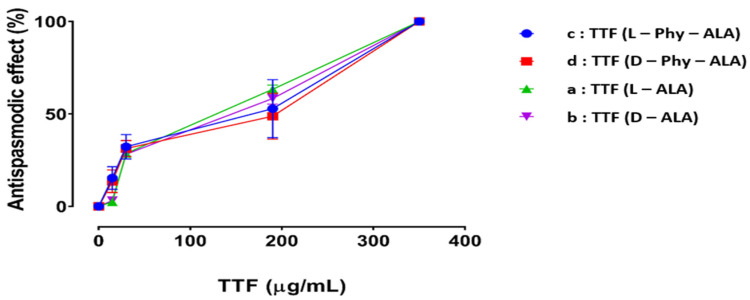
Antispasmodic effect of 2-thioxo-1,3-dithiol- carboxamides derivatives on Ach (10^−5^ M) induced spasm contraction of rabbit jejunum. Values are means ± SEM from 5 independent experiments, as shown.

When the spasm was induced by ACh (10 µM), as shown in Figure 3, all synthesis compounds reduced the jejunum’s contraction in a concentration-dependent manner, reaching 100% relaxation at the highest concentration (350 μg/mL) for all 2-thioxo-1,3-dithiol derivatives containing carboxamides compounds (**TDTCAs**) examined. In the experiments, the IC50s for D-Phy-ALA, L-phy-ALA, D-ALA and L-ALA, respectively, were 61.3, 68.9, 81.56 and 89.30 μg/mL, suggesting that the D configuration enhances antispasmodic activity compared to the L configuration. Additionally, the results indicate that phenyl groups were active groups in 2-thioxo-1,3-dithiol-carboxamides (**TDTCAs**) tested compounds. 

### 2.5. Molecular Docking Studies

Our study tested compounds **a**, **b**, **c** and **d** on jejunum isolated from rabbits, evaluating their antispasmodic effects on ACh-induced contractions. All compounds demonstrated significant antispasmodic activity, with compounds **c** and **d** exhibiting the most pronounced effects. These results suggest potential applications of these compounds in treating gastrointestinal disorders characterized by spasms. Understanding the mechanism behind these effects is crucial for further development and therapeutic use. Molecular docking is an effective method for determining the interaction types and binding sites of molecules with target proteins [26,27,28,29,30]. Given the lack of information in the literature regarding our specific molecules and their potential targets, we selected a range of receptors to identify the best targets. These receptors included the M3 muscarinic receptor, the beta-2 adrenergic receptor, the alpha-adrenergic receptor, and the L-type calcium channel. The docking scores of the compounds with M3 Muscarinic Acetylcholine (4DAJ), the Cav1.2 (L-type VGCC) receptor (6JP5), beta-adrenergic receptor (2R4R), and alpha-adrenergic receptor (7VFS) were calculated using MOE modeling software. The results are summarized in Table 1.

**Figure 4:**
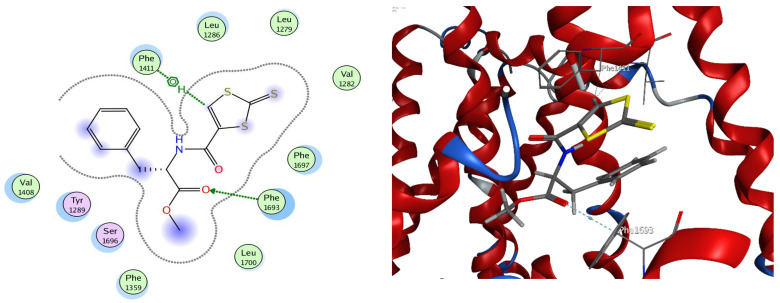
2D and 3D docking of compound **d** and alpha-adrenergic receptor.

The alpha-adrenergic receptor emerged as the best target protein, followed by voltage-dependent calcium channels, for our synthesized molecules. Among the tested compounds, compound **d** showed the highest binding affinity with the alpha-adrenergic receptor, scoring −6.47 kcal/mol, followed by compound **c** with a score of −6.27 kcal/mol. Both compounds **c** and **d** also demonstrated good binding affinity with the Cav1.2 receptor (Table 2). The binding affinity with muscarinic and beta-adrenergic receptors was less significant. This finding is particularly relevant as it explains the potent antispasmodic effects observed experimentally. The interactions with alpha-adrenergic receptors might modulate adrenergic signaling pathways, while the binding to calcium channels promotes relaxation by reducing calcium influx in the intestine. We hypothesize that the antispasmodic effects of compounds **c** and **d** may be due to the inhibition of both alpha-adrenergic receptors and calcium channels. Additionally, it is plausible that inhibition of alpha-adrenergic receptors could lead to downstream inhibition of calcium channels, further contributing to muscle relaxation. To elucidate the binding mechanism, we focused on the interaction between the ligands and the amino acids within the protein binding pocket, examining the types of bonds formed in the complexes of compound **d** (see Figure 4).

For compound d, our analysis revealed that the side chain residues of Phe1693 form hydrogen bonds as donors with the carbonyl groups of the compound. Additionally, there is a significant π-π stacking interaction between Phe1411 and the dithio-2-thione ring of the ligand. In summary, the combined experimental and docking data provide a comprehensive understanding of the antispasmodic effects of compounds **c** and **d**. These interactions underscore the potential of these compounds as therapeutic agents for gastrointestinal disorders involving hypermotility and spasms.

Table 3 lists the chemicals and chosen protein coenzymes with which hydrogen bonds can form. Figure 4 shows the poses that the enzyme-calmed compounds, specifically the alpha-adrenergic receptor (7VFS), assumed that were most appropriate.

In order to identify a precise docking study between the drugs and the target proteins, MOE stands as the primary molecular docking method used. A high docking score for compound **d** was obtained by hydrogen π-stacking with the six-membered ring of PHE 1411, which resulted in an interaction distance of 3.65 Å, energy stabilization of −0.8 kcal/mol, and through oxygen atom to the PHE 1693 amino acid residue. With an energy stabilization of −1.6 kcal/mol, this hydrogen bonding is approximately 3.03 Å distant. 

The target receptor’s structure was stabilized by the bonds formed with the important amino acid residues of the binding pockets that were discovered based on the studies discussed above. Due to the strong affinity of all docked poses with the lowest binding energy, they are all considered to be in the best-docked conformation. The docking program (MOE) also made it feasible to match the experimentally observed binding modes, thereby defining the specific conformation of the target and ligand, in light of these crucial molecular interactions.

## 3. Experimental

### 3.1. General Methods

The various reagents such as ethylene trithiocarbonate 97% and dimethyl acetylenedicarboxylate (DMAD) 99%, hexafluorophosphate benzotriazoletetramethyluronium (HBTU) 98%, triethylamine 99.5%, tetrabutylammonium bromide (TBAB) 99% and amino acids 99% (L-alanine, D-alanine, L-phenylalanine, D-phenylalanine), triethylamine 99.5%, thionyl chloride (SOCl_2_) 98% and amino acids 99%, sodium hydroxide (NaOH) 99.99%, ethyl acetate (HPLC-grade) hexane (HPLC-grade), etrahydrofuran (HPLC-grade), toluene (HPLC-grade) and dichloromethane (HPLC-grade) were purchased from Sigma-Aldrich. Silica gel 60 from Merck was used for column liquid chromatography (230–400 mesh ASTM). For thin-layer chromatography (TLC), Merck aluminum plates covered with Merck silica gel 60 F254 (0.2 mm thick) were employed. An ultraviolet lamp (UV) set at 254 nm was used to reveal the chemicals that had been produced. A digital Electrothermal IA series 9000 fusiometer using capillary tubes was used to calculate their melting point. A Bruker Corporation, Billerica, MA, USA, Ascend 400 MHz-Advance III HD NMR spectrometer was used to record the NMR analyses. Additionally, ^13^C and ^1^H NMR spectra were captured at 100 and 400 MHz, respectively, using CDCl_3_ as solvent. The chemical shifts of the various peaks were given in ppm, and the coupling constants (J) were given in Hz. To characterize the various signals, the following abbreviations were used: singlet (s), doublet (d), doublet (dd), multiplet (m) and quadruplet (q). At the mass spectrometry service of the University of Valencia in Spain, the high-resolution mass spectra (ESI-TOF-MS) were recorded in the EI (70 eV) or FAB mode and were given as *m*/*z* (% of relative intensity).

### 3.2. Synthesis of 2-Thioxo-1,3-dithiol-4,5-dicarboxylic Acid Dimethyl Ester (***4***)

Dimethyl acetylenedicarboxylate (10.4 g, 0.073 mol) and ethylene trithiocarbonate (10 g, 0.073 mol) were dissolved in 25 mL of toluene. The mixture was heated to reflux at 150 °C overnight [19]. After cooling the mixture in a water/ice bath, the product precipitated out, was filtered without washing, and dried under vacuum, yielding 16.8 g of the product.

### 3.3. Synthesis of 2-Thioxo-1,3-dithiol-4,5-dicarboxylic Acid (***5***)

A mixture containing 1 mole of 2-Thioxo-1,3-dithiol-4,5-dicarboxylic acid dimethyl ester and 1.2 moles of sodium hydroxide in 30 mL of THF/water solution (1/2: *v*/*v*) was stirred at room temperature for 24 h. Unreacted ester was separated from the aqueous solution by three extractions with dichloromethane (DCM) after evaporating the THF [19]. A precipitate formed by evaporating half of the aqueous phase and adding 3 M HCl, and the resulting crude product was extracted through simple filtration.

### 3.4. Procedure for the Preparation of Carboxylic Acids Groups Derived from Amino Acids (***7***)

At 0 °C, 2 moles of SOCl_2_ were slowly introduced into methanol (MeOH). After 15 min, this mixture was combined with 1 mole of amino acid, stirred for 2 h at 25 °C, and subsequently refluxed for an additional 2 h. Excess unreacted MeOH and SOCl_2_ were removed using a rotary evaporator. The remaining residue was dissolved in MeOH, and diethyl ether was added until precipitation occurred, followed by filtration [20,21,22,23].

### 3.5. Procedure for the Preparation of 2-Thioxo-1,3-dithiol-carboxamides (***a***–***d***)

A solution containing hexafluorophosphate benzotriazole tetramethyluronium (HBTU, 1.1 mol) and 2-thioxo-1,3-dithiol-4,5-dicarboxylic acid (1 mol) in dichloromethane (DCM, 30 mL) was stirred at room temperature for 10 min. Subsequently, a solution of protected amino acid (2.2 mol) was added to the mixture, followed by the gradual addition of triethylamine (TEA, 3.3 mol) over a period of 15 min at 0 °C. The reaction was then allowed to stir at room temperature for 1 h [20,21,22,23].

**Compound a**: (R)-methyl 2-(2-thioxo-1,3-dithiol-4-carboxamido)propanoate 

Yield (%) = 81; mp = 391 K; Rf: 0.71 (hexane/ethyl acetate: 4:1 *v*/*v*), **^1^H NMR (400 MHz, CDCl_3_)**: 1.5 (d, 3H, CH-CH_3_, J = 7.2); 3.8 (s, 3H, CO_2_-CH_3_); 4.6 (m, 1H, CHq); 6.7 (s, 1H, HCS); 7.6 (s,1H,CO-NH). **^13^C NMR (300 MHz, CDCl_3_)**: 18.64 (CH-CH_3_); 49.20 (CH-NH); 49.20 (CH_3-_CO_2_); 53.36 (Cq-CO); 132.52(CH-S); 157.33 (Cq-CO); 173,53 (Cq-CO_2_); 211.11 (Cq=S). Mass Spectrometry: [MH]+ *m/z =* 263.

**Compound b**: (S)-methyl 2-(2-thioxo-1,3-dithiol-4-carboxamido)propanoate 

Yield (%) = 87; mp = 394 K; Rf: 0.70 (hexane/ethyl acetate: 4:1 *v*/*v*), **^1^H NMR (400 MHz, CDCl_3_)**: 1.6 (d, 3H, CH-CH_3_, J = 7.2); 3.8 (s, 3H, CO_2_-CH_3_); 3.1 (m, 1H, CHq); 6.5 (s, 1H, HCS); 7.6 (s,1H,CO-NH). **^13^C NMR (300 MHz, CDCl_3_)**: 18.62 (CH-CH_3_); 49.09 (CH-NH); 49.19 (CH_3-_CO_2_); 53.36 (Cq-CO); 132.54(CH-S); 157.37 (Cq-CO); 173,57 (Cq-CO_2_); 211.17 (Cq=S). Mass Spectrometry: [MH]+ *m/z =* 263.

**Compound c**: (R)-methyl 3-phenyl-2-(2-thioxo-1,3-dithiol-4-carboxamido)propanoate 

Yield (%) = 84; mp = 429 K; Rf: 0.67 (hexane/ethyl acetate: 4:1 *v*/*v*), **^1^H NMR (400 MHz, CDCl_3_)**: 3.08 (2dd, 2H, CH_2_-C_6_H_5_, J = 6.6, 12.6); 3.69 (s, 3H, CO_2_-CH_3_); 4.84 (m, 1H, CHq); 6.70 (s, 1H, HCS); 6.88 (s,1H,CO-NH); 7.22 (m, 3H, 2CH-Ar)); 7.49 (m, 3H, 3CH-Ar). **^13^C NMR (300 MHz, CDCl_3_)**: 37.96 (CH_2_-C_6_H_5_); 53.26 (CH_3_-CO_2_); 54.20 (CH_-_NH); 127.92 (CH-C_6_H_5_); 129.26(2CH-C_6_H_5_); 129.57 (2CH-C_6_H_5_); 132.78 (CH-CO); 135.75 (Cq-C_6_H_5_); 140.35 (CH-S); 157.56 (Cq-CONH); 172.21 (Cq-CO-CO_2_); 211.32 (Cq-C=S). Mass Spectrometry: [MH]+ *m/z =* 240.

**Compound d**: (S)-methyl 3-phenyl-2-(2-thioxo-1,3-dithiol-4-carboxamido)propanoate 

Yield (%) = 89; mp = 422 K; Rf: 0.69 (hexane/ethyl acetate: 4:1 *v*/*v*), **^1^H NMR (400 MHz, CDCl_3_)**: 3.08 (2dd, 2H, CH_2_-C_6_H_5_, J = 6.3, 18.3); 3.7 (s, 3H, CO_2_-CH_3_); 4.8 (m, 1H, CHq); 6.68 (s, 1H, HCS); 7.15 (s,1H,CO-NH); 7.23 (m, 3H, 2CH-Ar)); 47.47 (m, 3H, 3CH-Ar). **^13^C NMR (300 MHz, CDCl_3_)**: 37.968 (CH_2_-C_6_H_5_); 53.26 (CH_3_-CO_2_); 54.27 (CH_-_NH); 127.93 (CH-C_6_H_5_); 129.27(2CH-C_6_H_5_); 129.56 (2CH-C_6_H_5_); 132.73 (CH-CO); 135.69 (Cq-C_6_H_5_); 140.30 (CH-S); 157.55 (Cq-CONH); 172.15 (Cq-CO-CO_2_); 211.22 (Cq-C=S). Mass Spectrometry: [MH]+ *m/z =* 240.

### 3.6. Computational Method

The calculations were performed by the density functional theory (DFT) implemented in the Gaussian 09 package [27,28,29,30]. All geometries of intermediates, transition states and resulted products were optimized with a B3LYP/6-31G(d,p) base site in the gas phase [31,32,33]. In addition, the HOMO-LUMO energy and Molecular Electrostatic Potential (MEP) were determined with the same method and visualized by the GaussView 6.0.16 program [27].

### 3.7. Antispasmodic Activity

#### 3.7.1. Animals

These experiments were carried out on both female and male New Zealand rabbits (2–2.5 kg, 3 months). The animals were retained in a room with an air-conditioner, controlled lighting (12 h/12 h light/darkness cycle), with free access to food and water, in the animal house of the faculty of sciences and technology, fez, Morocco. Food was taken out 24 h before the experiment. All animals were taken care of in compliance with the internationally accepted guide for the care and use of laboratory animals published by the US National Institutes of Health [34].

#### 3.7.2. Tissue Preparation

Rabbits were anesthetized; the abdominal cavity was then opened, fragments of jejunum were dissected and cut into a length of about 1 cm, and then the piece of jejunum was fixed vertically in an isolated organ bath (10 mL volume) filled with Krebs–Henseleit buffer (KHS). Its lower end was attached to a tissue holder in the organ bath and the upper end was connected to an isometric force transducer (UF1 Isometric Transducer) connecting to a polygraph, it was maintained under a tension of 1 g. The KHS was maintained at 37 °C and continuously oxygenated with a carbogen mixture (95% O_2_ + 5% CO_2_). The tissue preparations were left under 1 g tension for at least 30 min until stabilization. After stabilization, segments with weak contractions were removed from the protocol. For each test, we used a new segment of the jejunum.

#### 3.7.3. Experimental Protocol

After a stabilization period (30 min), the eventual antispasmodic effect of 2-thioxo-1,3-dithiol-carboxamides derivatives (0.15–350 μg/mL) was tested directly on spontaneous rabbit jejunum contractions. In order to assess the spasmolytic effect of our products, the spasm was induced by acetylcholine (ACh, 10^−5^ M). Once the plateau of contraction elicited by spasmogen was achieved, 2-thioxo-1,3-dithiol-carboxamides derivatives (**a**–**d**) were added to the organ bath cumulatively (0.15–350 μg/mL).

### 3.8. Molecular Docking Studies

The Molecular Operating Environment (MOE v2009) was used to determine and report the scores of the molecular docking technique. ChemDraw (18.2) was used to create the atomic structures. Using the Protein Data Bank, the target crystal structures for the alpha-adrenergic receptor (PDB code = 7VFS), Beta-adrenergic receptor (PDB code = 2R4R), muscarinic receptor M3 (PDB code = 4DAJ) and L-type calcium channel (PDB code = 6JP5) were found and created [26]. All the cofactors and ligands that bind to water were removed from the protein structure in order to optimize it, and then the structure was fixed with hydrogen atoms. Only the active sites were removed, to create phony atoms. It was altered to stand for the MMFF94x force field with all of its attributes and charges. Setting up the alpha site spheres required the use of the MOE site search module, and docking the structural models of the molecules to the surface of the cancer protein within required the use of the MOE dock module. The upgrading was then carried out utilizing two unconnected refining procedures. The dock scoring in the MOE program was carried out using the London dG scoring algorithm. Following study, self-turning docks were permitted in the top ten dock poses that received the highest grades. The database browser was then used to match the docking postures with the ligand in the co-crystallized structure, allowing the docking pose‘s RMSD to be calculated. The binding free energy and hydrogen bonds between the created molecules and the receptors’ amino acid residues were then calculated in order to classify the binding affinity of the compounds to the studied protein molecules. The RMSD of the (native) ligand in the receptor structure was used to develop the default-docking model, which includes interaction types similar to those.

## 4. Conclusions

Synthesis of 2-thioxo-1,3-dithiol-carboxamides (**TDTCAs**) was achieved by coupling 2-thioxo-1,3-dithiol-4,5-dicarboxylic acid with various amino acids, using HBTU as the coupling agent. This process resulted in the formation of 2-thioxo-1,3-dithiol-carboxamides compounds (**a**–**d**) by establishing amide bonds through the coupling of the prepared thiones. Following synthesis, all products underwent purification using silica gel column chromatography. The structures of these compounds were determined using analytical methods including ^1^H and ^13^C NMR spectroscopy and high-resolution mass spectrometry (HRMS). Furthermore, the HOMO-LUMO energy gap was determined using DFT calculations at the B3LYP/6−31G(d,p) level. The results revealed that compound **d** was more stable than its analogs. According to a study of four 2-thioxo-1,3-dithiol-carboxamides (**TDTCAs**), the majority of products tested had antispasmodic activity, with configuration **d** having the greatest antispasmodic activity. This compound could be used as a lead for the development of new therapeutic agents, including drugs to treat disorders related to smooth muscle function. It is important to note that the goal of the docking investigation was to examine how the most potent drugs interacted with the target protein, 7VFS. The results support the experimental findings by demonstrating that interactions between hydrogen and pi-cations and the ester function have a significant impact on the activity values under study. Compound **d** was discovered to have the highest binding affinity to the target protein based on computational analysis. Based on the aforementioned information, this chemical may be proposed as a critical structure for the development and synthesis of new and potent medications.

## Figures and Tables

**Table 1:** Intermolecular docking energy values (kcal/mol) of compounds on various receptors.

Compounds	M3 (kcal/mol)	Cav1.2 (kcal/mol)	Beta-2 Adrenergic Receptor (kcal/mol)	Alpha Adrenergic Receptor (kcal/mol)
**a**	−3.96	−3.86	---	−5.02
**b**	−3.93	−4.24	---	−5.01
**c**	−4.86	−6.09	−4.05	−6.27
**d**	−5.32	−5.88	−4.56	−6.47

**Table 2:** Docking score and energy of the compounds and alpha-adrenergic receptor.

Compounds	S (kcal/mol)	Rmsd_Refine (kcal/mol)	E_Conf (kcal/mol)	E_Place (kcal/mol)	E_Score1 (kcal/mol)	E_Refine (kcal/mol)	E_Score2 (kcal/mol)
**a**	−5.02	4.08	3.36	−57.11	−7.01	−16.86	−5.02
**b**	−5.01	4.08	3.36	−57.11	−7.01	−16.86	−5.01
**c**	−6.27	2.04	29.20	−81.21	−8.21	−24.54	−6.27
	−6.19	1.94	26.80	−74.38	−8.24	−21.08	−6.19
	−6.16	1.88	47.78	−79.36	−8.25	−15.37	−6.16
	−6.15	1.68	32.46	−74.36	−7.96	−22.11	−6.15
	−6.14	2.18	26.92	−60.80	−8.02	−21.96	−6.14
**d**	**−6.47**	1.36	33.08	−53.93	−7.79	−22.45	−6.47
	−6.27	1.34	50.12	−59.75	−7.72	−14.22	−6.27
	−6.25	2.07	31.87	−54.93	−8.03	−21.80	−6.25
	−6.13	1.45	24.94	−71.34	−7.98	−24.25	−6.13
	−6.09	2.29	26.56	−72.24	−8.44	−22.74	−6.09

Key Interactions for Compound **d.** The bold indicate that it’s the best value.

**Table 3:** Interaction table between the compounds and alpha-adrenergic receptor (7VFS).

Compounds	Ligand	Receptor	Interaction	Distance (Å)	E (kcal/mol)
**c**	S5	SD MET 1293	H-donor	4.4	−0.4
	6-ring	6-ring PHE 1411	pi-pi	3.89	0
**d**	O29	CB PHE 1693	H-acceptor	3.03	−0.9
	C2	6-ring PHE 1411	H-pi	3.65	−0.8

## Data Availability

The original contributions presented in the study are included in the article/Supplementary Material, further inquiries can be directed to the corresponding authors.

## Supplementary Materials

The following supporting information can be downloaded at: https://www.mdpi.com/article/10.3390/molecules29163855/s1.
