# Supplementary material for: An Investigation of Novel Series of 2-Thioxo-1,3-dithiol-carboxamides as Potential Antispasmodic Agents: Design, Synthesis via Coupling Reactions, Density Functional Theory Calculations, and Molecular Docking
Source: Molecules. 2024 Aug 14;29(16):3855. doi: 10.3390/molecules29163855 (PMC11356991; doi:10.3390/molecules29163855)

**supporting information**

**NMR 1H Compound C : DTTF-D-PHe-ALA-OMe**

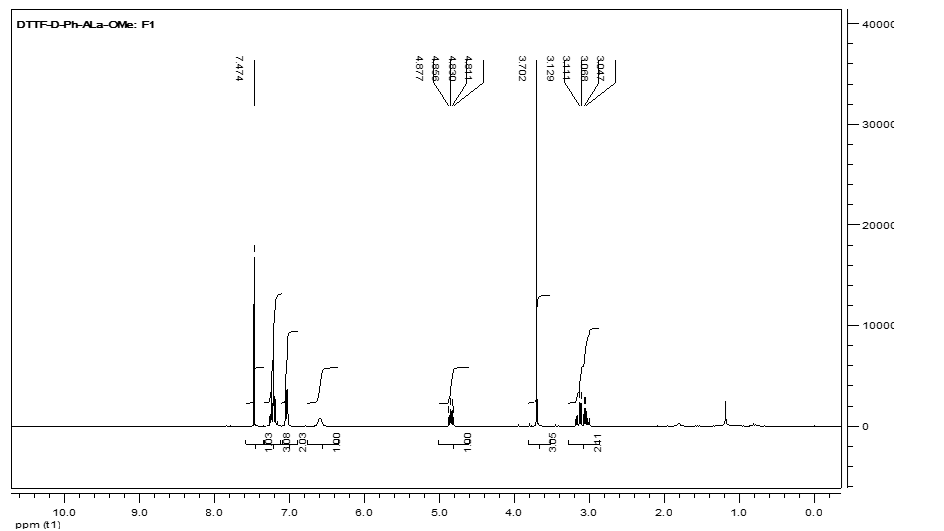


**NMR ^13^C : DTTF-D- PHe-ALA-OMe**


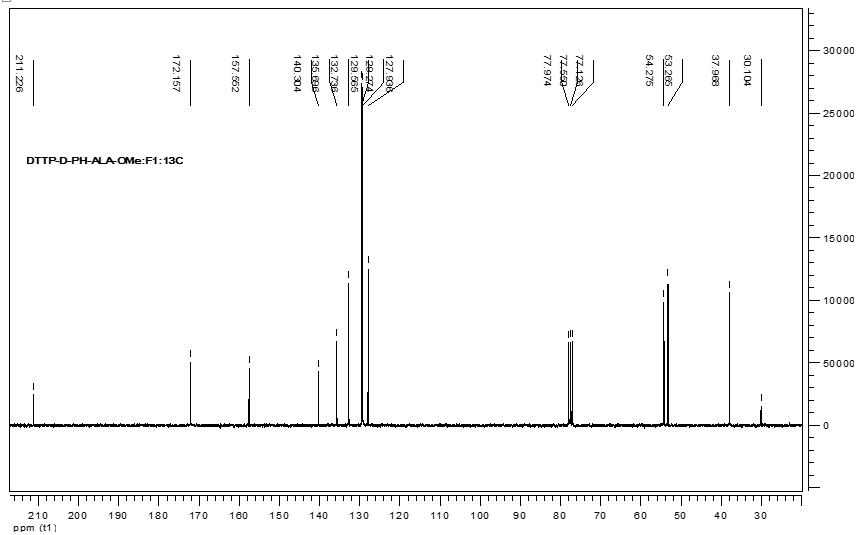


**HRMS : DTTF-D- PHe-ALA-OMe**


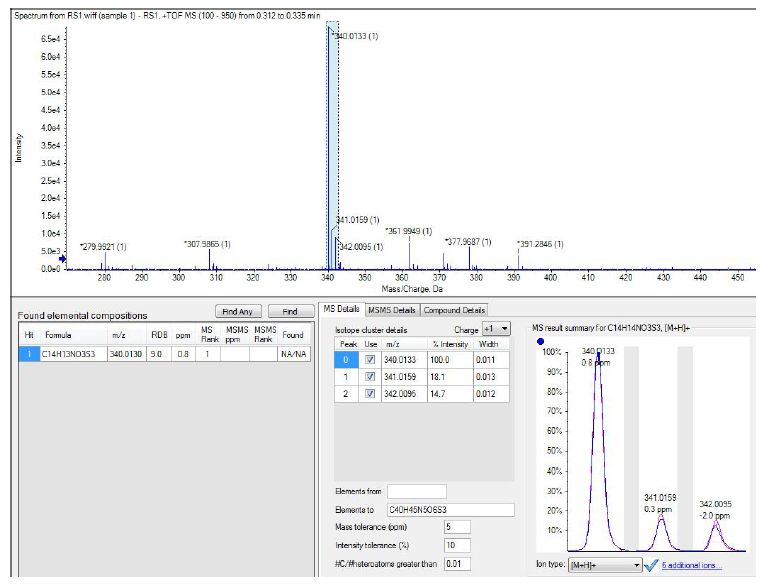


**NMR ^1^H : DTTF-L-PHe-ALA-OMe**

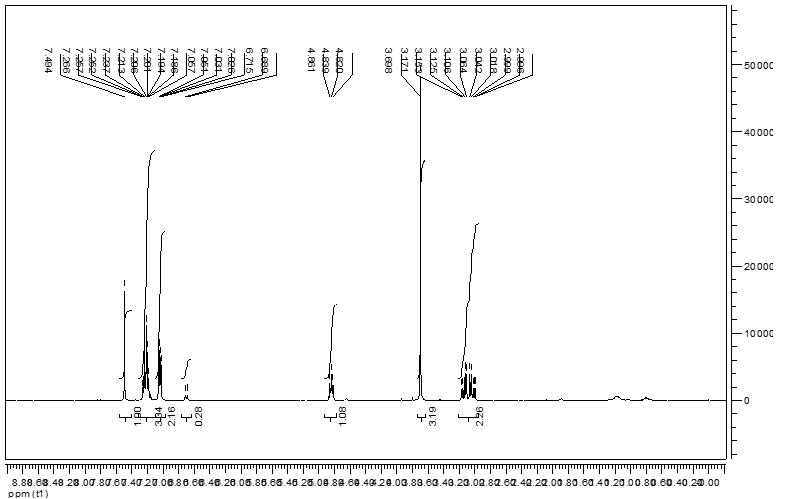


**NMR ^13^C : DTTF-L-PHe-ALA-OMe**


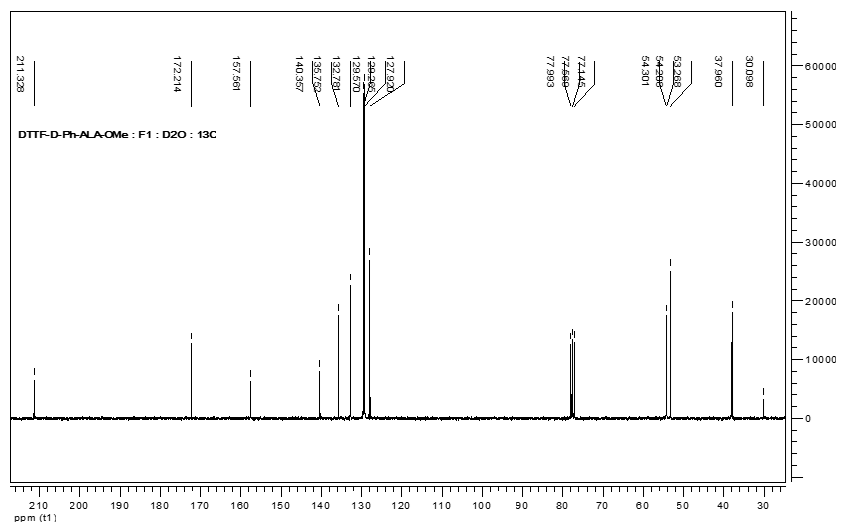


**HRMS : DTTF-L- PHe-ALA-OMe**


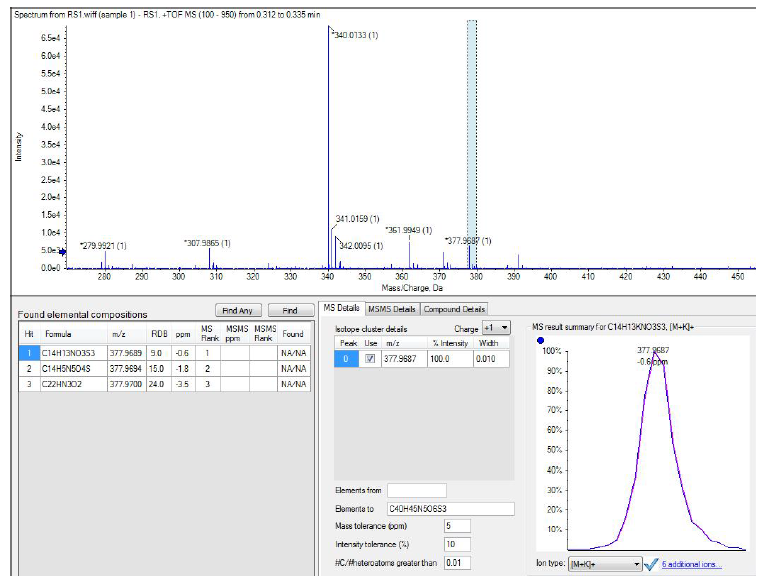


**NMR ^1^H Compound a : DTTF-L-ALA-OMe**

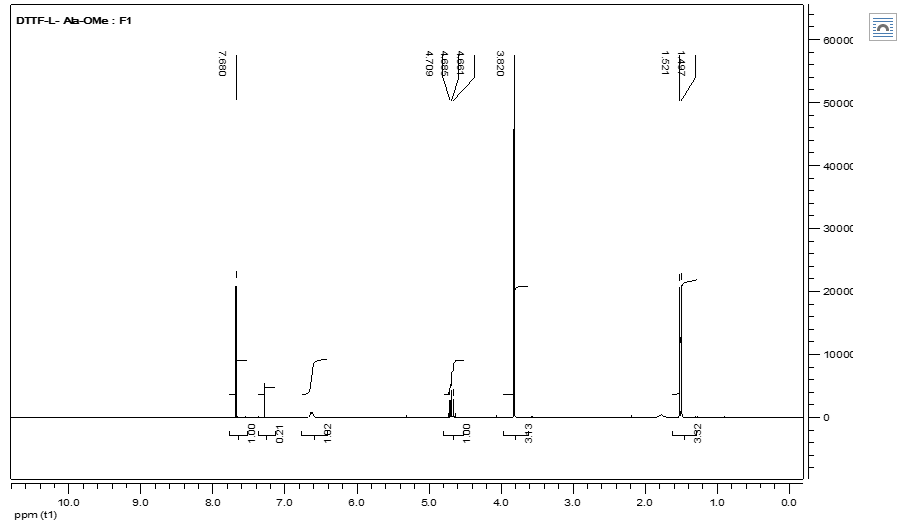


**NMR ^13^C : DTTF-L-ALA-OMe**


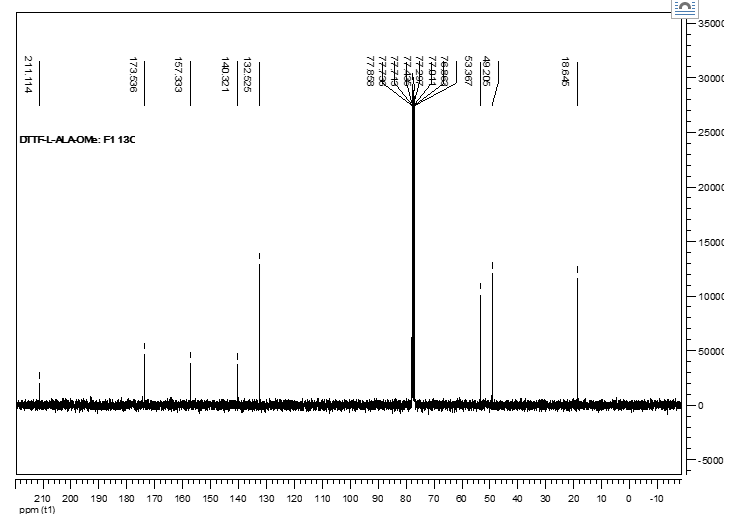


**HRMS : DTTF-L -ALA-OMe**


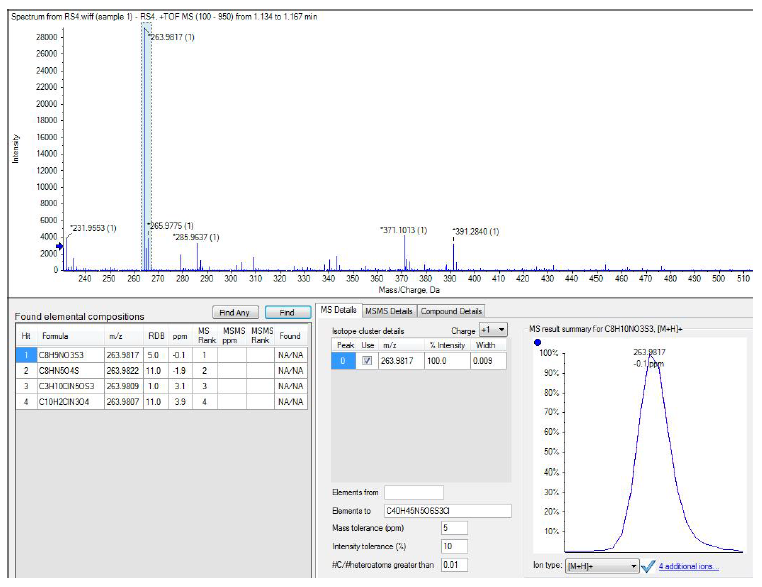


**NMR ^1^H : DTTF-D-ALA-OMe**

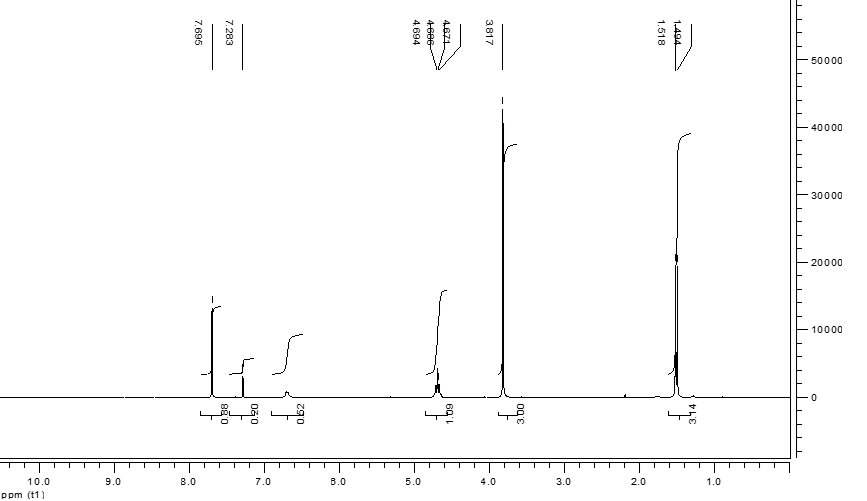


**NMR ^13^C : DTTF-D-ALA-OMe**


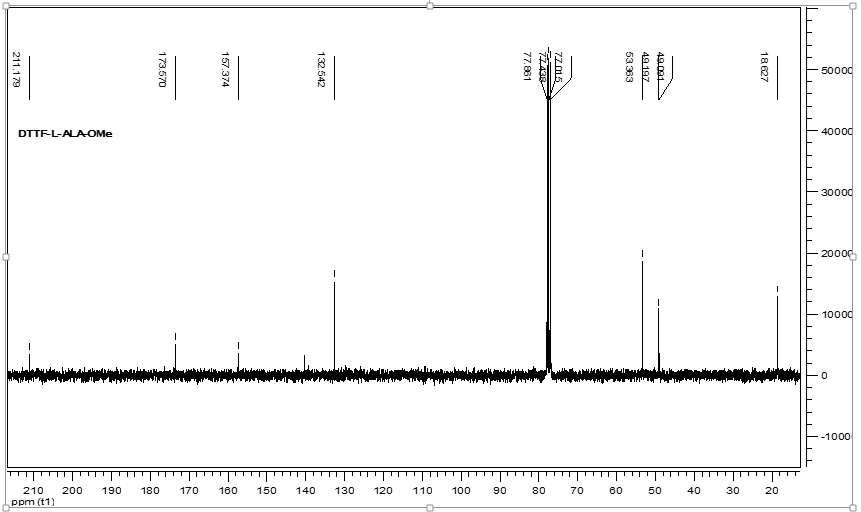


**HRMS : DTTF-D -ALA-OMe**


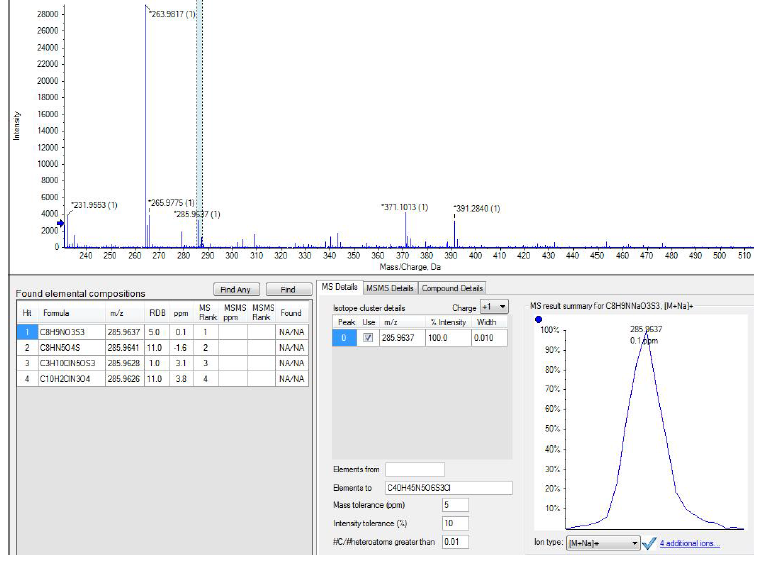

Supplement: Supplementary file 1 [file molecules-29-03855-s001.zip › molecules-3144644-supplementary.docx]
